# Risks of second primary cancer among patients with major histological types of lung cancers in both men and women

**DOI:** 10.1038/sj.bjc.6605616

**Published:** 2010-03-30

**Authors:** S-C Chuang, G Scélo, Y-C A Lee, S Friis, E Pukkala, D H Brewster, K Hemminki, E Tracey, E Weiderpass, S Tamaro, V Pompe-Kirn, E V Kliewer, K-S Chia, J M Tonita, C Martos, J G Jonasson, P Boffetta, P Brennan, M Hashibe

**Affiliations:** 1 International Agency for Research on Cancer (IARC), Lyon, France; 2 Department of Epidemiology and Biostatistics, School of Public Health, Imperial College London, London, UK; 3 Department of Epidemiology, School of Public Health, University of California, Los Angeles, CA, USA; 4 Institute of Cancer Epidemiology, Danish Cancer Society, Copenhagen, Denmark; 5 Finnish Cancer Registry, Institute for Statistical and Epidemiology Cancer Research, Helsinki, Finland; 6 Scottish Cancer Registry, Information Services, NHS National Services Scotland, Edinburgh, Scotland, UK; 7 Division of Molecular Genetic Epidemiology, German Cancer Research Center (DKFZ), Heidelberg, Germany; 8 Center for Family and Community Medicine, Karolinska Institutet, Huddinge, Sweden; 9 New South Wales Cancer Registry, Eveleigh, New South Wales, Australia; 10 The Cancer Registry of Norway, Oslo, Norway; 11 Department of Community Medicine, University of Tromso, Tromso, Norway; 12 Department of Medical Epidemiology and Biostatistics, Karolinska Institutet, Stockholm, Sweden; 13 Department of Genetic Epidemiology, Samfundet Folkhalsan, Helsinki, Finland; 14 British Columbia Cancer Agency, Vancouver, British Columbia, Canada; 15 Cancer Registry of Slovenia, Institute of Oncology, Ljubljana, Slovenia; 16 Epidemiology and Cancer Registry, CancerCare Manitoba, Winnipeg, Manibota, Canada; 17 Department of Community Health Sciences, University of Manitoba, Winnipeg, Manibota, Canada; 18 Center for Molecular Epidemiology, Singapore; 19 Singapore Cancer Registry, Singapore; 20 Saskatchewan Cancer Agency, Regina, Saskatchewan, Canada; 21 Cancer Registry of Zaragoza, Aragon Health Science Institute, Zaragoza, Spain; 22 Icelandic Cancer Registry, Icelandic Cancer Society, Reykjavik, Iceland; 23 Faculty of Medicine, University of Iceland, Reykjavik, Iceland; 24 The Tisch Cancer Institute, Mount Sinai School of Medicine, New York, NY, USA; 25 International Prevention Research Institute, Lyon, France; 26 University of Utah School of Medicine, Salt Lake City, UT, USA

**Keywords:** lung cancer, SPCs, sex differences

## Abstract

**Background:**

Patterns of second primary cancers (SPCs) following first primary lung cancers (FPLCs) may provide aetiological insights into FPLC.

**Methods:**

Cases of FPLCs in 13 cancer registries in Europe, Australia, Canada, and Singapore were followed up from the date of FPLC diagnosis to the date of SPC diagnosis, date of death, or end of follow-up. Standardised incidence ratios (SIRs) were calculated to estimate the magnitude of SPC development following squamous cell carcinoma (SCC), small cell lung carcinoma (SCLC), and adenocarcinoma (ADC).

**Results:**

Among SCC patients, male SIR=1.58 (95% confidence interval (CI)=1.50–1.66) and female SIR=2.31 (1.94–2.72) for smoking-related SPC. Among SCLC patients, the respective ratios were 1.39 (1.20–1.60) and 2.28 (1.73–2.95), and among ADC patients, they were 1.73 (1.57–1.90) and 2.24 (1.91–2.61). We also observed associations between first primary lung ADC and second primary breast cancer in women (SIR=1.25, 95% CI=1.05–1.48) and prostate cancer (1.56, 1.39–1.79) in men.

**Conclusion:**

The FPLC patients carried excess risks of smoking-related SPCs. An association between first primary lung ADC and second primary breast and ovarian cancer in women at younger age and prostate cancers in men may reflect an aetiological role of hormones in lung ADC.

Lung cancer is the most common cancer in the world, representing 12.4% of all new cancer cases in the year 2002 (Parkin *et al*, 2005), its main risk factor being tobacco smoking. Other risk factors are involuntary smoking and occupational exposure to agents such as asbestos, radon, arsenic, and silica dust; suspected risk factors include air pollution and dietary factors, notably low consumption of fruits and vegetables (Teppo *et al*, 2001; Spitz *et al*, 2006; World Cancer Research Fund, 2007).

There are four major lung cancer subtypes: squamous cell carcinoma (SCC), large cell carcinoma, adenocarcinoma (ADC), and small cell lung carcinoma (SCLC), comprising over 90% of all cases (Spitz *et al*, 2006). Although smoking is associated with all histological types of lung cancer, ADC has a weaker association with smoking than other types in terms of smoking status, intensity, duration, age at start, dose, and years since quitting (Barbone *et al*, 1997).

Several studies examined second primary cancers (SPCs) following lung cancer (Levi *et al*, 1999; Teppo *et al*, 2001; Liu *et al*, 2002; Duchateau and Stokkel, 2005), but not by gender. As lung cancer epidemiology is different in men and women, aetiology may also differ and be reflected in SPCs. This study includes three major histological types of lung cancer: SCC, SCLC, and ADC. We have examined differences in the pattern of SPCs between men and women who had a first primary lung cancer (FPLCs).

## Materials and methods

An international multicentre study was initiated to incorporate large cancer registries that have been in operation for at least 25 years, to conduct a systematic analysis of SPCs (Brennan *et al*, 2005; Scelo *et al*, 2006). The registries included New South Wales in Australia, British Columbia, Manitoba and Saskatchewan in Canada, Denmark, Finland, Iceland, Norway, Scotland, Singapore, Slovenia, Sweden, and Zaragoza in Spain. These registries had cancer data covering different time periods within the period 1943–2000. A high degree of ascertainment completeness by the registry is suggested by consistent inclusion in subsequent volumes of Cancer Incidence in Five Continents (Parkin *et al*, 2002).

Data were provided by each cancer registry on all first primary cancers, including age and sex of each subject, diagnosis and date of the first primary cancer, follow-up for mortality, and diagnosis and date of the SPCs, if any. In this study, any different cancer codes used by registries were systematically converted into International Classification of Disease, Ninth Revision (ICD-9). We analysed the occurrence of SPC in survivors of lung cancer (ICD-9: 162). Coding of multiple primaries in the cancer registries followed a common set of rules proposed by the International Association of Cancer Registries and the International Agency for Research on Cancer (IARC) (Muir and Percy, 1991). These define primary cancer as one that originates in a primary site or tissue and is thus neither an extension nor a recurrence nor a metastasis. Only one tumour was recognised as arising in an organ or pair of organs or tissue as defined by the three-character category of the ICD or the topography of the ICD-O. Non-melanoma skin cancer (ICD-9: 173) was excluded because of the inconsistency in reporting across registries. We defined smoking-related cancers according to the most recent IARC monograph on the subject as cancers of the lip (ICD-9: 140), tongue (141), salivary gland (142), mouth (143–145), oropharynx (146), nasopharynx (147), hypopharynx (148), pharynx unspecified (149), oesophagus (150), stomach (151), liver (155), pancreas (157), nose and nasal cavity (160), larynx (161), bladder (188), and kidney (189), and as leukaemia (204–208) (IARC Working Group, 2004).

All cases of FPLC were followed up from the date of the first lung cancer diagnosis (1943–2000) to the date of SPC (1943–2000), date of death, or end of follow-up (1992–2000). To assess the potential excess occurrence of SPC, the numbers of SPCs observed and expected were used to estimate the standardised incidence ratios (SIRs). The SIRs adjusted for age, sex, year, and registry were calculated using indirect standardisation methods (Brennan *et al*, 2005). The expected number was calculated from accumulated person-years and age-, sex-, and calendar period-specific cancer incidence rates in each of the cancer registries.

We used Poisson regression to model the observed and expected number and estimate the gender effect on SIRs (Richiardi *et al*, 2007). The cumulative risk of second smoking- or non-smoking-related cancers was calculated by taking into account death as a competing risk (Gooley *et al*, 1999). The absolute excess risk (AER) – a measure for estimating the absolute burden or magnitude of a health problem – was defined as the difference between the observed and expected number of SPCs divided by the total number of person-years at risk, and is expressed as per 100 000 person-years (Travis, 2006).

## Results

Table 1 summarises the characteristics of patients with a primary lung cancer by histology. A total of 63 609 females with 100 036 person-years of observation and 194 950 males with 311 666 person-years of observation were identified across the 13 cancer registries. The mean follow-up periods were 1.6 years for both men and women. During the follow-up, a total of 5383 SPCs were recorded. Among the 258 559 lung cancers, 3259 SCCs (2.4%), 557 SCLCs (1.0%), and 1567 ADCs (2.2%) developed an SPC, indicating an excess risk of SPCs of 36% for women and 25% for men (data not shown).

Table 2 shows the SIR and AER of the selected SPCs by sex and histology. The detailed SIRs for each SPC site and by histology are listed in Appendix Table A1. The SIRs for smoking-related second cancers were 2.31 (95% confidence interval (CI)=1.94–2.72) for female and 1.58 (95% CI=1.50–1.66) for male SCC patients; 2.28 (95% CI=1.73–2.95) for female and 1.39 (95% CI=1.20–1.60) for male SCLC patients; and 2.24 (95% CI=1.91–2.61) for female and 1.73 (95% CI=1.57–1.90) for male ADC patients. In addition to the relative measures, the absolute measure, AERs, were higher in smoking-related cancers than in non-smoking-related cancers in SCC and SCLC, whereas the burden was almost the same in ADC. Associations with second primary breast cancer (SIR=1.25, 95% CI=1.05–1.48) among women, prostate cancer (SIR=1.56, 95% CI=1.39–1.79) among men, and second primary thyroid cancer among both men (SIR=3.43, 95% CI=1.57–8.62) and women (SIR=4.87, 95% CI=2.88–7.69) were observed after first primary lung ADC (*P*-value for heterogeneity among three histological types <0.05); such associations were not observed for lung SCC and SCLC.

**Figure 1:**
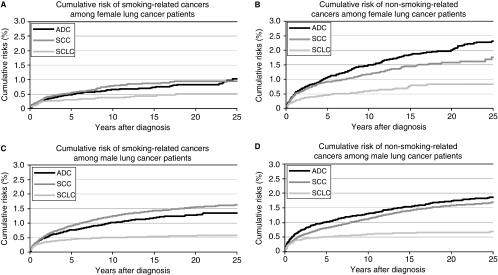
Cumulative risks of selected second primary cancer following a first primary lung cancer, taking death into account as the competing risk. ADC, adenocarcinoma; SCC, squamous cell lung carcinoma; SCLC, small cell carcinoma. **A**: smoking-related SPCs among female patients; **B**: non-smoking-related SPCs among female patients; **C**: smoking-related SPCs among male patients; **D**: non-smoking-related SPCs among male patients.

Figure 1 shows the observed cumulative risk for smoking- and non-smoking-related SPCs by sex and histology, taking death into account as a competing risk. The censored and event numbers are presented in Appendix Table A2. The 25-year cumulative risks among SCLC patients were low: among women, 0.51% developed smoking-related SPCs and 0.84% developed non-smoking-related SPCs; among men, the rates were 0.58 and 0.70%, respectively. Among SCC and ADC cases, men had higher smoking-related SPC risk than women: 1.64% (95% CI=1.54–1.73) *vs* 0.96% (95% CI=0.78–1.13) among SCC patients and 1.35% (95% CI=1.21–1.50) *vs* 1.02% (95% CI=0.80–1.24) among ADC patients. In terms of non-smoking-related SPCs, women and men had similar 25-year cumulative risks (1.77%, 95% CI=1.46–2.08 for women and 1.69%, 95% CI=1.60–1.79 for men) among SCC cases, but women had a higher cumulative risk among ADC cases (2.32%, 95% CI=2.03–2.60 for women and 1.87%, 95% CI=1.69–2.05 for men).

**Figure 2:**
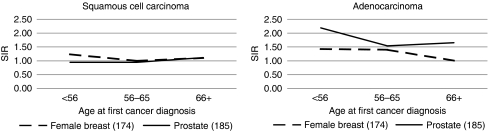
Standardised incidence ratios (SIRs) of second primary breast cancer in women and prostate cancer in men by age at diagnosis of first primary lung adenocarcinoma and squamous cell carcinoma.

Figure 2 presents the SIRs of second primary female breast and prostate cancer by age at diagnosis of the first primary lung ADC and SCC. We observed associations with second primary breast cancer for those whose first primary lung ADC was diagnosed at younger age (SIR=1.43, 95% CI=1.03–1.92 for <56 years of age; and SIR=1.40, 95% CI=1.04–1.84 for 56–65 years of age). Such an association was not observed among cases of first primary lung SCC. The associations between ADC and second primary prostate cancer were present in all age strata but decreased slightly in the older age groups. There were similar associations with ovarian cancer for women diagnosed with first primary lung ADC below 56 years of age (observed *N*=12, SIR=2.25, 95% CI=1.16–3.94); second primary testicular cancer was associated with first primary lung ADC only among those who were 56–65 years of age at first cancer registration (observed *N*=3).

Appendix Table A3 lists SIRs for selected SPCs by calendar period and histology. Overall, the SIRs for smoking-related SPCs for women were 2.33 (95% CI=1.73–3.07) before 1975, 2.37 (95% CI=1.94–2.86) between 1975 and 1983, 2.35 (95% CI=1.95–2.81) between 1984 and 1990, and 2.03 (95% CI=1.62–2.52) after 1990; the corresponding SIRs within the same calendar period for men were 1.44 (95% CI=1.31–1.58), 1.59 (95% CI=1.47–1.71), 1.63 (95% CI=1.40–1.77), and 1.72 (95% CI=1.55–1.91), respectively.

## Discussion

In our study, 2% of individuals with primary lung cancer developed an SPC during a mean follow-up period of 1.6 years, representing excess risks of 36% for women and 25% for men. Overall, we observed excess risks of smoking-related SPCs for both genders in all three histological types of FPLC, as well as some types of non-smoking-related SPCs with first primary lung ADC.

The relative excess risks of smoking-related cancers after lung cancer were stronger in women than in men. Although lung cancer incidence is increasing in women, incidence rates of most smoking-related cancers are lower in women than in men. For example, the incidence rate per 100 000 of head and neck cancer in the year 2002 was 15.3 in men and 4.6 in women (Ferlay *et al*, 2004), the difference reflecting the lower incidence of primary smoking-related cancers in women in the reference general population; comparison of AERs shows a larger AER in men.

We observed associations between smoking-related SPCs and first primary lung SCC and SCLC for both men and women. For ADC, associations were observed not only with smoking-related cancers but also with other cancers. ADC accounted for 31–54% of lung cancers among male non-smokers and for 49–74% of lung cancers among female non-smokers in North America (Charloux *et al*, 1997), implying the involvement of non-smoking factors in its aetiology. We observed increased SIRs for second primary female breast and ovarian cancer after lung ADC to be more pronounced among young women. Such an association was not noted in first primary lung SCC and SCLC; in men, an association between prostate and lung ADC was also observed. Because breast, ovary, and prostate cancers are hormone-related cancers, hormone-related factors may have a role in lung ADC aetiology.

A negative association between endometrial cancer and lung SCC and SCLC was observed (but not with lung ADC) (Table 2), which is consistent with its inverse association with cigarette smoking but positive association with oestrogens (Cook *et al*, 2006). Although IARC has reclassified colorectal cancer as being smoking related (Secretan *et al*, 2009), no association was observed between first primary lung and second primary colorectal cancers. Given the long induction period of smoking in colorectal carcinogenesis and the decreased risk after the cessation of smoking (Secretan *et al*, 2009), and the fact that quitting smoking is usual among lung cancer patients, this was expected. However, in contrast to most of the studies (Liang *et al*, 2009) that found a stronger association with smoking in rectal than in colon cancers, we observed an association between lung ADC and colon cancer, but not with rectal cancer. Smoking has been consistently associated with colorectal adenomatous polyps, a precancerous lesion of colorectal cancer (Botteri *et al*, 2008), which might suggest that ADCs share common risk factors. Nevertheless, such a pattern was not observed in the association between lung ADC and oesophageal ADC.

In our study, the SIRs for smoking-related SPCs after a first primary SCC and SCLC did not change significantly across the calendar years, but we observed an increasing trend for overall smoking-related SPCs in male ADC patients (*P* for trend=0.04), suggesting a time trend between tobacco smoking and a more important role in the development of ADC; this was not observed in female ADC cases (*P*=0.74).

The median life expectancy in untreated SCLC is about 6 to 12 weeks (Schiller, 2002). In our study, 75% of SCLC patients were followed up for less than 1 year and the main reason for terminating follow-up was death (97%). Fortunately, the large sample size across the registries enabled us to explore the SPCs for SCLC survivors who showed an excess risk for smoking-related, but not for non-smoking-related, cancers.

A major limitation of our study is the lack of individual-based smoking information. On the assumption that multiple cancers share risk factors, we separated smoking and non-smoking-related SPCs to examine the potential importance of smoking on male and female SPC development. We could not exclude the possibility of misclassification between metastasis and second primaries because we did not have relevant pathological information. However, our data were from high-quality cancer registries, 85% or over being microscopically verified (Parkin *et al*, 2002). In addition, the stratified analysis by follow-up years (data not shown) showed relatively stable SIRs across all strata. We surmised that the probability of an SPC being a metastasis would decrease with longer follow-up time. Individuals with FPLC may have increased surveillance for other smoking-related cancers, but the increased risks of non-smoking-related sites may reflect a more general surveillance bias. The large size of our study provided precision in stratified analyses.

In conclusion, SCC, SCLC, and ADC were associated with smoking-related SPCs in both men and women. Associations of ADC in female patients at younger age with second primary breast and ovarian cancers and of ADC in male patients with prostate cancer may reflect a role of hormones in lung ADC aetiology.

## Figures and Tables

**Table 1:** Descriptive characteristics of first primary lung cancer patients from 13 cancer registries

	**Squamous cell carcinoma**				**Small cell carcinoma**				**Adenocarcinoma excluding bronchioalveolar**			
	**Women**		**Men**		**Women**		**Men**		**Women**		**Men**	
	***N***	**%**	***N***	**%**	***N***	**%**	***N***	**%**	***N***	**%**	***N***	**%**
Total	20 468		113 197		15 639		37 663		27 502		44 090	
												
*Age at diagnosis (year)*												
<56	2990	14.61	15 128	13.36	2865	18.32	6577	17.46	6903	25.10	8528	19.34
56–65	6021	29.42	37 568	33.19	5188	33.17	12 960	34.41	8088	29.41	14 395	32.65
66–74	7184	35.10	40 731	35.98	5227	33.42	12 553	33.33	7669	27.89	14 225	32.26
75+	4273	20.88	19 770	17.47	2359	15.08	5573	14.80	4842	17.61	6942	15.75
												
*Calendar period at diagnosis*												
∼1975	1966	9.61	25 719	22.72	555	3.55	4590	12.19	2779	10.10	5444	12.35
1975–1983	4933	24.10	33 279	29.40	3371	21.56	10 399	27.61	5428	19.74	10 350	23.47
1984–1990	6318	30.87	29 442	26.01	5278	33.75	11 958	31.75	8175	29.73	13 281	30.12
1991∼	7251	35.43	24 757	21.87	6435	41.15	10 716	28.45	11 120	40.43	15 015	34.06
												
*Follow-up period*												
<12 months	13 342	65.18	69 243	61.17	11 168	71.41	28 757	76.35	17 899	65.08	20 704	46.96
1–4 year	5408	26.42	33 111	29.25	3921	25.07	7963	21.14	7129	25.92	10 260	23.27
5–9 year	959	4.69	5833	5.15	298	1.91	518	1.38	1407	5.12	1817	4.12
10+ year	759	3.71	5010	4.43	252	1.61	425	1.13	1067	3.88	1309	2.97
												
*Registries*												
Australia, NSW (1972–1997)	2076	10.14	10 893	9.62	2032	12.99	5076	13.48	2517	9.15	5331	12.09
Canada, British Columbia (1970–1998)	2313	11.30	7169	6.33	2092	13.38	3173	8.42	3617	13.15	4499	10.20
Canada, Manitoba (1970–1998)	675	3.30	3004	2.65	649	4.15	1241	3.30	1180	4.29	1766	4.01
Canada, Saskatchewan (1967–1998)	409	2.00	1924	1.70	267	1.71	760	2.02	571	2.08	783	1.78
Denmark (1943–1997)	3891	19.01	21 299	18.82	2926	18.71	5682	15.09	5762	20.95	7611	17.26
Finland (1953–1998)	1386	6.77	19 914	17.59	1177	7.53	8283	21.99	2027	7.37	5221	11.84
Iceland (1955–2000)	152	0.74	340	0.30	173	1.11	224	0.59	324	1.18	289	0.66
Norway (1953–1999)	1591	7.77	10 374	9.16	1875	11.99	4695	12.47	3032	11.02	5333	12.10
Singapore Chinese (1968–1992)	504	2.46	2697	2.38	185	1.18	793	2.11	994	3.61	1550	3.52
Slovenia (1961–1998)	303	1.48	5853	5.17	207	1.32	1073	2.85	774	2.81	1551	3.52
Spain, Zaragoza (1978–1998)	56	0.27	1975	1.74	17	0.11	696	1.85	101	0.37	635	1.44
Sweden (1961–1998)	2223	10.86	12 023	10.62	0	0.00	0	0.00	3971	14.44	5535	12.55
UK, Scotland (1975–1996)	4889	23.89	15 732	13.90	4039	25.83	5967	15.84	2632	9.57	3986	9.04

**Table 2:** Numbers of cases (Obs), standardised incidence ratio (SIR) and absolute excess risk (AER) per 100 000 person-years of selected second primary cancers after lung cancer by sex and histology

	**Squamous cell carcinoma**				**Small cell carcinoma**				**Adenocarcinoma excluding bronchioalveolar**			
**Cancer sites (ICD 9th revision)**	**Obs**	**SIR**	**95% CI**	**AER**	**Obs**	**SIR**	**95% CI**	**AER**	**Obs**	**SIR**	**95% CI**	**AER**
*Women*	**PY=34 344**				**PY=16 763**				**PY=48 929**			
All but non-melanoma skin	377	1.28	(1.16–1.42)	242.55	149	1.16	(0.98–1.36)	119.65	555	1.49	(1.37–1.62)	374.30
Smoking-related cancers	142	2.31	(1.94–2.72)	234.27	58	2.28	(1.73–2.95)	194.47	164	2.24	(1.91–2.61)	185.80
Head and neck	31	5.69	(3.87–8.08)	74.41	9	3.49	(1.59–6.63)	38.31	13	1.85	(0.99–3.17)	12.23
Oesophagus (150)	14	3.31	(1.81–5.56)	28.45	6	3.30	(1.21–7.18)	24.95	7	1.72	(0.69–3.55)	6.01
Bladder and kidney	40	2.31	(1.65–3.14)	65.95	18	2.45	(1.45–3.88)	63.59	69	3.32	(2.58–4.21)	98.59
Leukaemias (204–208)	9	1.28	(0.58–2.42)	5.73	9	3.06	(1.40–5.82)	36.14	22	2.50	(1.57–3.79)	27.00
Other smoking-related cancers	37	1.94	(1.36–2.67)	52.14	10	1.45	(0.70–2.67)	18.58	41	1.93	(1.38–2.62)	40.34
Colorectal (153, 154)	41	0.84	(0.60–1.14)	−22.74	14	0.67	(0.36–1.12)	−41.14	74	1.26	(0.99–1.58)	31.21
Colon (153)	30	0.90	(0.61–1.29)	−9.71	9	0.63	(0.29–1.19)	−31.53	56	1.43	(1.08–1.86)	34.42
Rectum (154)	11	0.70	(0.35–1.24)	−13.73	5	0.75	(0.24–1.74)	−9.94	18	0.93	(0.55–1.46)	−2.77
Non-smoking-related cancer	194	1.06	(0.91–1.22)	31.03	77	0.93	(0.74–1.16)	−33.66	317	1.32	(1.18–1.47)	157.14
Female breast (174)	87	1.09	(0.88–1.35)	20.92	29	0.77	(0.52–1.11)	−51.68	135	1.25	(1.05–1.48)	55.12
Cervix uteri (180)	11	1.31	(0.65–2.35)	7.58	6	1.56	(0.57–3.41)	12.85	12	1.07	(0.55–1.87)	1.63
Corpus uteri (182)	8	0.43	(0.19–0.85)	−30.88	3	0.36	(0.07–1.05)	−31.82	19	0.76	(0.46–1.18)	−12.52
Ovary (183)	23	1.47	(0.93–2.21)	21.41	9	1.28	(0.59–2.43)	11.74	28	1.37	(0.91–1.98)	15.52
Thyroid gland (193)	6	2.48	(0.91–5.40)	10.43	3	2.64	(0.54–7.71)	11.12	18	4.87	(2.88–7.69)	29.23
Other non-smoking-related cancers	59	1.01	(0.76–1.30)	1.57	27	1.10	(0.72–1.60)	14.12	105	1.47	(1.20–1.77)	68.16
												
*Men*	**PY=212 428**				**PY=34 293**				**PY=64 945**			
All but non-melanoma skin	2882	1.21	(1.16–1.25)	231.99	408	1.16	(1.05–1.27)	160.26	1012	1.47	(1.38–1.56)	498.00
Smoking-related cancers	1454	1.58	(1.50–1.66)	250.63	186	1.39	(1.20–1.60)	151.44	428	1.73	(1.57–1.90)	277.11
Head and neck	340	2.53	(2.27–2.81)	96.76	33	1.50	(1.04–2.11)	32.26	77	2.01	(1.59–2.51)	59.57
Oesophagus (150)	93	1.78	(1.44–2.18)	19.18	12	1.46	(0.75–2.55)	11.03	27	1.91	(1.26–3.09)	19.76
Bladder and kidney	565	1.87	(1.72–2.03)	123.83	70	1.62	(1.26–2.04)	77.98	159	1.90	(1.62–2.22)	116.16
Leukaemias (204–208)	74	0.98	(0.77–1.23)	−0.71	14	1.29	(0.71–2.17)	9.18	33	1.58	(1.09–2.37)	18.62
Other smoking-related cancers	382	1.07	(0.96–1.18)	11.56	57	1.14	(0.87–1.48)	21.01	132	1.45	(1.21–1.72)	62.99
Colorectal (153, 154)	395	1.01	(0.92–1.12)	1.84	46	0.79	(0.58–1.05)	−35.66	129	1.15	(0.96–1.38)	25.91
Colon (153)	223	1.00	(0.87–1.14)	0.00	29	0.85	(0.57–1.22)	−14.92	81	1.24	(0.98–1.57)	24.14
Rectum (154)	172	1.04	(0.89–1.21)	3.11	17	0.70	(0.41–1.13)	−21.25	48	1.02	(0.76–1.36)	1.45
Non-smoking-related cancer	1033	0.96	(0.90–1.02)	−20.48	176	1.09	(1.21–1.58)	44.48	455	1.39	(1.26–1.52)	195.41
Prostate (185)	694	1.06	(0.98–1.14)	18.49	111	1.19	(0.98–1.43)	51.68	312	1.56	(1.39–1.79)	172.37
Testis (186)	5	1.10	(0.36–2.56)	0.21	1	1.26	(0.03–6.99)	0.60	5	3.14	(1.01–9.55)	5.25
Thyroid gland (193)	10	1.14	(0.55–2.10)	0.58	4	3.04	(0.83–7.79)	7.83	9	3.43	(1.57–8.62)	9.82
Other non-smoking-related cancers	324	0.79	(0.71–0.88)	−39.77	60	0.92	(0.70–1.18)	−15.63	129	1.04	(0.87–1.24)	7.98

Abbreviations: CI=confidence interval; ICD=International Classification of Disease; Obs=observed number; PY=person-years.

Smoking-related cancers included: head and neck (ICD-9: lip: 140, tongue: 141, mouth: 143–145, oropharynx: 146, hypopharynx: 148, pharynx unspecified: 149, and larynx: 161), salivary gland (142), nasopharynx (147), oesophagus (150), nose and nasal cavity (160), stomach (151), liver (155), and pancreas (157), bladder and kidney (188 189), leukaemia (204–208).

AER is expressed as per 100 000.
