# Supplementary material for: Risks of second primary cancer among patients with major histological types of lung cancers in both men and women
Source: Br J Cancer. 2010 Mar 30;102(7):1190–5. doi: 10.1038/sj.bjc.6605616 (PMC2853101; doi:10.1038/sj.bjc.6605616)
Supplement: Supplementary Appendix Tables [file 6605616x1.doc]

Appendix Table 1. Numbers of cases (Obs) and standardized incidence ratios (SIR) of second primary cancers after a first primary lung cancer by histology

|  | **Squamous cell carcinoma** | | | | | |
| --- | --- | --- | --- | --- | --- | --- |
| Cancer sites (ICD 9th.revision) | Obs | SIR | 95% CI | Obs | SIR | 95% CI |
| Sex | Women | | | Men | | |
| All Malignant (140-208) | 410 | 1.28 | (1.16-1.41) | 3207 | 1.23 | (1.18-1.27) |
| All but non-melanoma skin | 377 | 1.28 | (1.16-1.42) | 2882 | 1.21 | (1.16-1.25) |
| Head and Neck (140, 141, 143-145, 146, 148, 149, 161) | 31 | 5.69 | (3.87-8.08) | 340 | 2.53 | (2.27-2.81) |
| Oral cavity, pharynx (140-149) | 14 | 2.63 | (1.44-4.41) | 179 | 1.87 | (1.61-2.17) |
| Lip (140) | 1 | 1.25 | (0.03-6.95) | 33 | 0.90 | (0.62-1.26) |
| Tongue (141) | 1 | 1.01 | (0.03-5.65) | 28 | 2.37 | (1.58-3.43) |
| Salivary gland (142) | 0 | 0.00 | (0.00-5.85) | 4 | 0.66 | (0.18-1.69) |
| Mouth (143-145) | 9 | 5.70 | (2.61-10.8) | 42 | 2.52 | (1.82-3.41) |
| Pharynx (146-149) | 3 | 2.25 | (0.46-6.58) | 72 | 2.95 | (2.31-3.72) |
| Oropharynx (146) | 0 | 0.00 | (0.00-6.78) | 30 | 3.15 | (2.13-4.50) |
| Nasopharynx (147) | 1 | 2.99 | (0.07-16.7) | 6 | 1.22 | (0.45-2.65) |
| Hypopharynx (148) | 1 | 2.94 | (0.07-16.4) | 29 | 3.44 | (2.30-4.94) |
| Pharynx unspecified (149) | 1 | 8.79 | (0.22-49.0) | 7 | 4.56 | (1.83-9.40) |
| Oesophagus (150) | 14 | 3.31 | (1.81-5.56) | 93 | 1.78 | (1.44-2.18) |
| Stomach (151) | 21 | 1.74 | (1.08-2.66) | 226 | 1.11 | (0.97-1.26) |
| Small intestine (152) | 1 | 1.07 | (0.03-5.96) | 24 | 2.94 | (1.88-4.37) |
| Colorectal (153,154) | 41 | 0.84 | (0.60-1.14) | 395 | 1.01 | (0.92-1.12) |
| Colon (153) | 30 | 0.90 | (0.61-1.29) | 223 | 1.00 | (0.87-1.14) |
| Rectum (154) | 11 | 0.70 | (0.35-1.24) | 172 | 1.04 | (0.89-1.21) |
| Liver, Gallbladder, bile ducts (155-156)(-155.2) | 6 | 0.83 | (0.30-1.80) | 68 | 1.09 | (0.85-1.38) |
| Liver (155)(-155.2) | 5 | 1.91 | (0.62-4.47) | 42 | 1.09 | (0.78-1.47) |
| Gallbladder, bile ducts (156) | 1 | 0.22 | (0.01-1.20) | 26 | 1.10 | (0.72-1.61) |
| Pancreas (157) | 19 | 1.68 | (1.01-2.62) | 88 | 0.90 | (0.72-1.11) |
| Peritoneum (158) | 1 | 1.70 | (0.04-9.49) | 1 | 0.32 | (0.01-1.79) |
| Nose and nasal cavity (160) | 2 | 3.76 | (0.45-13.6) | 16 | 2.44 | (1.40-3.97) |
| Larynx (161) | 18 | 16.60 | (9.85-26.3) | 171 | 3.44 | (2.95-4.00) |
| Bone (170) | 0 | 0.00 | (0.00-10.9) | 3 | 0.85 | (0.18-2.50) |
| Soft tissue sarcoma (171) | 3 | 2.25 | (0.46-6.58) | 13 | 1.04 | (0.55-1.77) |
| Melanoma of skin (172) | 5 | 0.58 | (0.19-1.35) | 44 | 0.72 | (0.52-0.96) |
| Other neoplasm of skin (173) | 33 | 1.24 | (0.85-1.74) | 325 | 1.49 | (1.34-1.66) |
| Female breast (174) | 87 | 1.09 | (0.88-1.35) | 0 |  |  |
| Male breast (175) | 0 |  |  | 6 | 1.31 | (0.48 -2.86) |
| Cervix uteri (180) | 11 | 1.31 | (0.65-2.35) | 0 |  |  |
| Placenta (181) | 0 | 0.00 | (0.00-0966) | 0 |  |  |
| Endometrium (182) | 8 | 0.43 | (0.19-0.85) | 0 |  |  |
| Ovary (183) | 23 | 1.47 | (0.93-2.21) | 0 |  |  |
| Other female genital (179,184) | 3 | 0.83 | (0.17-2.42) | 0 |  |  |
| Prostate (185) | 0 |  |  | 694 | 1.06 | (0.98-1.14) |
| Testis (186) | 0 |  |  | 5 | 1.10 | (0.36-2.56) |
| Other male genital (187) | 0 |  |  | 9 | 1.07 | (0.49-2.03) |
| Bladder (188,189.3,189.4) | 15 | 1.62 | (0.91-2.68) | 345 | 1.61 | (1.45-1.79) |
| Kidney (189)(-189.3,189.4) | 25 | 3.09 | (2.00-4.56) | 220 | 2.51 | (2.19-2.87) |
| Eye (190) | 2 | 2.53 | (0.31-9.15) | 4 | 0.61 | (0.16-1.55) |
| Brain, nervous system (191-192) | 6 | 1.44 | (0.53-3.13) | 22 | 0.66 | (0.41-1.00) |
| Thyroid gland (193) | 6 | 2.48 | (0.91-5.40) | 10 | 1.14 | (0.55-2.10) |
| Other endocrine gland (194,164.0) | 0 | 0.00 | (0.00-11.7) | 3 | 1.12 | (0.23-3.26) |
| Lymphohaematopoietic (200-208) | 30 | 1.28 | (0.87-1.83) | 174 | 0.87 | (0.75-1.01) |
| Lymphomas (200-202) | 16 | 1.41 | (0.80-2.28) | 75 | 0.92 | (0.72-1.15) |
| Hodgkin disease (201) | 2 | 2.49 | (0.30-8.98) | 9 | 1.01 | (0.46-1.91) |
| Non Hodgkin lymphoma (200,202) | 14 | 1.32 | (0.72-2.22) | 66 | 0.90 | (0.70-1.15) |
| Multiple myeloma (203) | 5 | 1.01 | (0.33-2.37) | 25 | 0.60 | (0.39-0.88) |
| Leukemias (204-208) | 9 | 1.28 | (0.58-2.42) | 74 | 0.98 | (0.77-1.23) |
| Lymphoid leukemia (204) | 4 | 1.27 | (0.35-3.26) | 36 | 0.96 | (0.67-1.33) |
| Myeloid leukemia (205) | 3 | 1.14 | (0.24-3.33) | 28 | 1.12 | (0.74-1.62) |
| Other leukemia (206-208) | 2 | 1.57 | (0.19-5.68) | 10 | 0.78 | (0.37-1.43) |
| Other malignant than defined | 16 | 1.00 | (0.57-1.62) | 69 | 0.56 | (0.44-0.71) |

Appendix Table 1. Continued

|  | **Small cell carcinoma** | | | | | |
| --- | --- | --- | --- | --- | --- | --- |
| Cancer sites (ICD 9th.revision) | Obs | SIR | 95% CI | Obs | SIR | 95% CI |
| Sex | Women | | | Men | | |
| All Malignant (140-208) | 159 | 1.13 | (0.96-1.31) | 445 | 1.16 | (1.05-1.27) |
| All but non-melanoma skin | 149 | 1.16 | (0.98-1.36) | 408 | 1.16 | (1.05-1.27) |
| Head and Neck (140, 141, 143-145, 146, 148, 149, 161) | 9 | 3.49 | (1.59-6.63) | 33 | 1.44 | (1.04-2.11) |
| Oral cavity, pharynx (140-149) | 9 | 3.71 | (1.70-7.04) | 18 | 1.16 | (0.69-1.84) |
| Lip (140) | 1 | 2.96 | (0.07-16.5) | 3 | 0.56 | (0.12-1.64) |
| Tongue (141) | 1 | 2.15 | (0.05-12.0) | 6 | 2.91 | (1.07-6.34) |
| Salivary gland (142) | 0 | 0.00 | (0.00-13.7) | 0 | 0.00 | (0.00-3.89) |
| Mouth (143-145) | 5 | 6.82 | (2.21-15.9) | 6 | 2.14 | (0.78-4.65) |
| Pharynx (146-149) | 2 | 3.22 | (0.39-11.6) | 3 | 0.70 | (0.14-2.04) |
| Oropharynx (146) | 0 | 0.00 | (0.00-13.3) | 2 | 1.19 | (0.14-4.31) |
| Nasopharynx (147) | 1 | 7.69 | (0.19-42.9) | 1 | 1.13 | (0.03-6.29) |
| Hypopharynx (148) | 1 | 6.38 | (0.16-35.6) | 0 | 0.00 | (0.00-2.58) |
| Pharynx unspecified (149) | 0 | 0.00 | (0.00-64.6) | 0 | 0.00 | (0.00-12.0) |
| Oesophagus (150) | 6 | 3.30 | (1.21-7.18) | 12 | 1.46 | (0.75-2.55) |
| Stomach (151) | 7 | 1.53 | (0.61-3.15) | 30 | 1.09 | (0.74-1.56) |
| Small intestine (152) | 1 | 2.77 | (0.07-15.4) | 4 | 3.63 | (0.99-9.29) |
| Colorectal (153,154) | 14 | 0.67 | (0.36-1.12) | 46 | 0.79 | (0.58-1.05) |
| Colon (153) | 9 | 0.63 | (0.29-1.19) | 29 | 0.85 | (0.57-1.22) |
| Rectum (154) | 5 | 0.75 | (0.24-1.74) | 17 | 0.70 | (0.41-1.13) |
| Liver, Gallbladder, bile ducts (155-156)(-155.2) | 2 | 0.77 | (0.09-2.79) | 9 | 1.02 | (0.47-1.93) |
| Liver (155)(-155.2) | 0 | 0.00 | (0.00-3.85) | 6 | 1.05 | (0.39-2.29) |
| Gallbladder, bile ducts (156) | 2 | 1.23 | (0.15-4.43) | 3 | 0.96 | (0.20-2.81) |
| Pancreas (157) | 8 | 1.75 | (0.76-3.45) | 19 | 1.38 | (0.83-2.15) |
| Peritoneum (158) | 0 | 0.00 | (0.00-13.4) | 0 | 0.00 | (0.00-7.62) |
| Nose and nasal cavity (160) | 0 | 0.00 | (0.00-15.7) | 1 | 1.04 | (0.03-5.79) |
| Larynx (161) | 1 | 1.82 | (0.05-10.1) | 16 | 1.94 | (1.11-3.15) |
| Bone (170) | 0 | 0.00 | (0.00-24.9) | 0 | 0.00 | (0.00-7.16) |
| Soft tissue sarcoma (171) | 0 | 0.00 | (0.00-6.89) | 6 | 3.31 | (1.22-7.21) |
| Melanoma of skin (172) | 1 | 0.22 | (0.01-1.21) | 5 | 0.42 | (0.14-0.99) |
| Other neoplasm of skin (173) | 10 | 0.85 | (0.41-1.56) | 37 | 1.21 | (0.85-1.67) |
| Female breast (174) | 29 | 0.77 | (0.52-1.11) | 0 |  |  |
| Male breast (175) | 0 |  |  | 0 | 0.00 | (0.00 -5.29) |
| Cervix uteri (180) | 6 | 1.56 | (0.57-3.41) | 0 |  |  |
| Placenta (181) | 0 | 0.00 | (0.00-1597) | 0 |  |  |
| Endometrium (182) | 3 | 0.36 | (0.07-1.05) | 0 |  |  |
| Ovary (183) | 9 | 1.28 | (0.59-2.43) | 0 |  |  |
| Other female genital (179,184) | 1 | 0.69 | (0.02-3.87) | 0 |  |  |
| Prostate (185) | 0 |  |  | 111 | 1.19 | (0.98-1.43) |
| Testis (186) | 0 |  |  | 1 | 1.26 | (0.03-6.99) |
| Other male genital (187) | 0 |  |  | 1 | 0.87 | (0.02-4.84) |
| Bladder (188,189.3,189.4) | 7 | 1.80 | (0.72-3.72) | 31 | 1.02 | (0.69-1.45) |
| Kidney (189)(-189.3,189.4) | 11 | 3.19 | (1.59-5.71) | 39 | 3.03 | (2.15-4.14) |
| Eye (190) | 0 | 0.00 | (0.00-10.3) | 2 | 2.04 | (0.25-7.36) |
| Brain, nervous system (191-192) | 2 | 1.01 | (0.12-3.64) | 6 | 1.06 | (0.39-2.31) |
| Thyroid gland (193) | 3 | 2.64 | (0.54-7.71) | 4 | 3.04 | (0.83-7.79) |
| Other endocrine gland (194,164.0) | 1 | 6.73 | (0.17-37.5) | 1 | 2.45 | (0.06-13.7) |
| Lymphohaematopoietic (200-208) | 21 | 2.09 | (1.29-3.20) | 38 | 1.29 | (0.91-1.77) |
| Lymphomas (200-202) | 10 | 1.97 | (0.95-3.63) | 19 | 1.49 | (0.90-2.33) |
| Hodgkin disease (201) | 0 | 0.00 | (0.00-10.6) | 1 | 0.79 | (0.02-4.40) |
| Non Hodgkin lymphoma (200,202) | 10 | 2.12 | (1.02-3.90) | 18 | 1.57 | (0.93-2.48) |
| Multiple myeloma (203) | 2 | 0.98 | (0.12-3.54) | 5 | 0.84 | (0.27-1.95) |
| Leukemias (204-208) | 9 | 3.06 | (1.40-5.82) | 14 | 1.29 | (0.71-2.17) |
| Lymphoid leukemia (204) | 3 | 2.29 | (0.47-6.70) | 3 | 0.57 | (0.12-1.65) |
| Myeloid leukemia (205) | 4 | 3.21 | (0.88-8.23) | 8 | 1.94 | (0.84-3.82) |
| Other leukemia (206-208) | 2 | 5.20 | (0.63-18.8) | 3 | 2.17 | (0.45-6.33) |
| Other malignant than defined | 7 | 1.08 | (0.43-2.22) | 8 | 0.42 | (0.18-0.83) |

Appendix Table 1. Continued

|  | **Adenocarcinoma excluding bronchioalveolar** | | | | | |
| --- | --- | --- | --- | --- | --- | --- |
| Cancer sites (ICD 9th.revision) | Obs | SIR | 95% CI | Obs | SIR | 95% CI |
| Sex | Women | | | Men | | |
| All Malignant (140-208) | 583 | 1.46 | (1.34-1.58) | 1074 | 1.44 | (1.36-1.55) |
| All but non-melanoma skin | 555 | 1.49 | (1.37-1.62) | 1012 | 1.47 | (1.38-1.56) |
| Head and Neck (140, 141, 143-145, 146, 148, 149, 161) | 13 | 1.85 | (0.99-3.17) | 77 | 2.01 | (1.59-2.51) |
| Oral cavity, pharynx (140-149) | 9 | 1.35 | (0.62-2.57) | 49 | 1.76 | (1.30-2.51) |
| Lip (140) | 0 | 0.00 | (0.00-24.6) | 10 | 1.05 | (0.50-1.96) |
| Tongue (141) | 1 | 0.82 | (0.01-4.55) | 8 | 2.18 | (0.94-5.13) |
| Salivary gland (142) | 1 | 1.22 | (0.02-6.79) | 1 | 0.58 | (0.01-2.86) |
| Mouth (143-145) | 3 | 1.58 | (0.32-4.60) | 12 | 2.35 | (1.21-4.92) |
| Pharynx (146-149) | 4 | 2.38 | (0.64-6.08) | 18 | 2.30 | (1.36-4.26) |
| Oropharynx (146) | 1 | 1.41 | (0.02-7.87) | 9 | 3.01 | (1.37-7.32) |
| Nasopharynx (147) | 0 | 0.00 | (0.00-54.4) | 2 | 1.17 | (0.13-4.37) |
| Hypopharynx (148) | 3 | 7.66 | (1.54-22.4) | 4 | 1.52 | (0.41-4.31) |
| Pharynx unspecified (149) | 0 | 0.00 | (0.00-192) | 3 | 5.99 | (1.2-24.81) |
| Oesophagus (150) | 7 | 1.72 | (0.69-3.55) | 27 | 1.91 | (1.26-3.09) |
| Stomach (151) | 24 | 1.65 | (1.06-2.45) | 62 | 1.27 | (0.97-1.67) |
| Small intestine (152) | 6 | 4.82 | (1.76-10.5) | 19 | 8.21 | (4.94-20.1) |
| Colorectal (153,154) | 74 | 1.26 | (0.99-1.58) | 129 | 1.15 | (0.96-1.38) |
| Colon (153) | 56 | 1.43 | (1.08-1.86) | 81 | 1.24 | (0.98-1.57) |
| Rectum (154) | 18 | 0.93 | (0.55-1.46) | 48 | 1.02 | (0.76-1.36) |
| Liver, Gallbladder, bile ducts (155-156)(-155.2) | 9 | 0.97 | (0.44-1.84) | 16 | 0.93 | (0.53-1.49) |
| Liver (155)(-155.2) | 6 | 1.85 | (0.68-4.03) | 13 | 1.19 | (0.63-2.10) |
| Gallbladder, bile ducts (156) | 3 | 0.50 | (0.10-1.45) | 3 | 0.47 | (0.09-1.16) |
| Pancreas (157) | 21 | 1.54 | (0.95-2.35) | 52 | 1.99 | (1.49-2.87) |
| Peritoneum (158) | 2 | 2.82 | (0.32-10.2) | 1 | 1.17 | (0.02-6.68) |
| Nose and nasal cavity (160) | 1 | 10.03 | (0.13-55.8) | 2 | 1.10 | (0.12-4.05) |
| Larynx (161) | 5 | 3.98 | (1.28-9.29) | 31 | 2.23 | (1.52-3.61) |
| Bone (170) | 2 | 4.53 | (0.51-16.4) | 2 | 2.14 | (0.24-9.05) |
| Soft tissue sarcoma (171) | 7 | 4.02 | (1.61-8.28) | 2 | 0.55 | (0.06-1.74) |
| Melanoma of skin (172) | 12 | 1.00 | (0.52-1.75) | 16 | 0.72 | (0.41-1.11) |
| Other neoplasm of skin (173) | 28 | 1.00 | (0.67-1.45) | 62 | 1.11 | (0.85-1.45) |
| Female breast (174) | 135 | 1.25 | (1.05-1.48) | 0 |  |  |
| Male breast (175) | 0 |  |  | 3 | 2.27 | (0.46-7.96) |
| Cervix uteri (180) | 12 | 1.07 | (0.55-1.87) | 0 |  |  |
| Placenta (181) | 0 | 0.00 | (0-2692.47) | 0 |  |  |
| Endometrium (182) | 19 | 0.76 | (0.46-1.18) | 0 |  |  |
| Ovary (183) | 28 | 1.37 | (0.91-1.98) | 0 |  |  |
| Other female genital (179,184) | 6 | 1.35 | (0.49-2.93) | 0 |  |  |
| Prostate (185) |  |  |  | 312 | 1.56 | (1.39-1.79) |
| Testis (186) |  |  |  | 5 | 3.14 | (1.01-9.55) |
| Other male genital (187) |  |  |  | 3 | 1.33 | (0.27-4.14) |
| Bladder (188,189.3,189.4) | 30 | 2.87 | (1.93-4.09) | 98 | 1.69 | (1.37-2.17) |
| Kidney (189)(-189.3,189.4) | 39 | 3.79 | (2.69-5.18) | 61 | 2.39 | (1.83-3.45) |
| Eye (190) | 0 | 0.00 | (0.00-24.4) | 3 | 1.59 | (0.32-5.17) |
| Brain, nervous system (191-192) | 11 | 2.03 | (1.01-3.63) | 14 | 1.32 | (0.72-2.34) |
| Thyroid gland (193) | 18 | 4.87 | (2.88-7.69) | 9 | 3.43 | (1.57-8.62) |
| Other endocrine gland (194,164.0) | 3 | 6.89 | (1.39-20.1) | 1 | 1.34 | (0.02-7.88) |
| Lymphohaematopoietic (200-208) | 41 | 1.42 | (1.02-1.93) | 66 | 1.14 | (0.88-1.47) |
| Lymphomas (200-202) | 11 | 0.78 | (0.39-1.39) | 25 | 0.99 | (0.64-1.46) |
| Hodgkin disease (201) | 0 | 0.00 | (0.00-24.9) | 1 | 0.42 | (0.01-1.96) |
| Non Hodgkin lymphoma (200,202) | 11 | 0.84 | (0.42-1.49) | 24 | 1.04 | (0.67-1.56) |
| Multiple myeloma (203) | 8 | 1.34 | (0.58-2.65) | 8 | 0.67 | (0.29-1.22) |
| Leukemias (204-208) | 22 | 2.50 | (1.57-3.79) | 33 | 1.58 | (1.09-2.37) |
| Lymphoid leukemia (204) | 8 | 2.07 | (0.89-4.09) | 18 | 1.77 | (1.05-3.11) |
| Myeloid leukemia (205) | 9 | 2.89 | (1.32-5.49) | 6 | 0.86 | (0.31-1.81) |
| Other leukemia (206-208) | 5 | 2.75 | (0.89-6.43) | 9 | 2.46 | (1.12-5.71) |
| Other malignant than defined | 34 | 1.85 | (1.28-2.58) | 29 | 0.82 | (0.55-1.14) |

Appendix Table 2. Numbers of censored and events by the histology of the first primary lung cancer by gender

| Years after diagnosis | 0 | 0-5 | 5-10 | 10-15 | 15-20 | 20-25 | >25 |
| --- | --- | --- | --- | --- | --- | --- | --- |
| **Female** |  |  |  |  |  |  |  |
| **ADC** |  |  |  |  |  |  |  |
| Survivors without SPC | 27502 | 27502 | 4701 | 3941 | 3715 | 3616 | 3563 |
| Smoking-related events | 0 | 118 | 27 | 7 | 6 | 5 | 1 |
| Non-smoking-related events | 0 | 259 | 69 | 36 | 10 | 13 | 4 |
| Death | 0 | 22424 | 664 | 183 | 83 | 35 | 18 |
| **SCC** |  |  |  |  |  |  |  |
| Survivors without SPC | 20468 | 20468 | 3094 | 2591 | 2369 | 2296 | 2282 |
| Smoking-related events | 0 | 102 | 29 | 7 | 4 | 0 | 0 |
| Non-smoking-related events | 0 | 172 | 32 | 21 | 6 | 3 | 1 |
| Death | 0 | 17100 | 442 | 194 | 63 | 11 | 13 |
| **SCLC** |  |  |  |  |  |  |  |
| Survivors without SPC | 15639 | 15639 | 1323 | 1148 | 1081 | 1066 | 1064 |
| Smoking-related events | 0 | 46 | 7 | 3 | 2 | 0 | 0 |
| Non-smoking-related events | 0 | 65 | 15 | 9 | 2 | 0 | 0 |
| Death | 0 | 14205 | 153 | 55 | 11 | 2 | 4 |
| **Male** |  |  |  |  |  |  |  |
| **ADC** |  |  |  |  |  |  |  |
| Survivors without SPC | 44090 | 44090 | 5354 | 4205 | 3767 | 3627 | 3569 |
| Smoking-related events | 0 | 309 | 68 | 32 | 11 | 6 | 2 |
| Non-smoking-related events | 0 | 419 | 80 | 46 | 22 | 10 | 7 |
| Death | 0 | 38008 | 1001 | 360 | 107 | 42 | 24 |
| **SCC** |  |  |  |  |  |  |  |
| Survivors without SPC | 37663 | 113197 | 14880 | 10686 | 8844 | 8091 | 7779 |
| Smoking-related events | 0 | 978 | 280 | 120 | 50 | 20 | 6 |
| Non-smoking-related events | 0 | 886 | 256 | 182 | 73 | 25 | 6 |
| Death | 0 | 96453 | 3658 | 1540 | 630 | 267 | 97 |
| **SCLC** |  |  |  |  |  |  |  |
| Survivors without SPC | 37663 | 37663 | 1862 | 1540 | 1421 | 1395 | 1381 |
| Smoking-related events | 0 | 160 | 17 | 7 | 1 | 1 | 0 |
| Non-smoking-related events | 0 | 190 | 22 | 7 | 1 | 1 | 1 |
| Death | 0 | 35451 | 283 | 105 | 24 | 12 | 5 |

SPC: Second primary cancer

ADC: Adenocarcinoma

SCC: Squamous cell carcinoma

SCLC: Small cell lung cancer

Appendix Table 3. Numbers of cases (Obs) and standardized incidence ratios (SIR) of selected second primary cancers after a first primary lung cancer by histology and years of first cancer diagnosis

|  | **Squamous cell carcinoma** | | | | | | | | | | | |
| --- | --- | --- | --- | --- | --- | --- | --- | --- | --- | --- | --- | --- |
| Period at first cancer registration | < 1975 | | | 1975 - 1983 | | | 1984 - 1990 | | | 1991 + | | |
| *Cancer sites (ICD 9th.revision)* | Obs | **SIR** | (95% CI) | Obs | **SIR** | (95% CI) | Obs | **SIR** | (95% CI) | Obs | **SIR** | (95% CI) |
| **Women** |  |  |  |  |  |  |  |  |  |  |  |  |
| All but non-melanoma skin | 51 | 1.59 | (1.19, 2.09) | 93 | 1.04 | (0.84, 1.27) | 132 | 1.36 | (1.14, 1.61) | 101 | 1.36 | (1.10, 1.65) |
| Smoking-related cancers | 20 | 2.75 | (1.68, 4.25) | 39 | 2.01 | (1.43, 2.75) | 49 | 2.42 | (1.79, 3.20) | 34 | 2.32 | (1.61, 3.24) |
| Head and neck | 6 | 10.32 | (3.77, 22.5) | 7 | 3.73 | (1.49, 7.68) | 10 | 4.83 | (2.31, 8.88) | 8 | 5.15 | (2.22, 10.2) |
| Oesophagus (150) | 1 | 2.83 | (0.07, 15.7) | 4 | 2.98 | (0.81, 7.63) | 6 | 4.10 | (1.50, 8.92) | 3 | 2.82 | (0.58, 8.23) |
| Bladder and kidney | 6 | 3.34 | (1.22, 7.28) | 16 | 3.02 | (1.73, 4.91) | 11 | 1.89 | (0.94, 3.39) | 7 | 1.57 | (0.63, 3.23) |
| Leukemias (204-208) | 2 | 2.52 | (0.30, 9.10) | 0 | 0.00 | (0.00, 1.65) | 4 | 1.72 | (0.47, 4.41) | 3 | 1.76 | (0.3 , 5.14) |
| Other smoking-related cancers | 5 | 1.33 | (0.43, 3.11) | 12 | 1.38 | (0.71, 2.41) | 18 | 2.11 | (1.25, 3.33) | 13 | 2.22 | (1.18, 3.79) |
| Colorectal (153, 154) | 3 | 0.59 | (0.12-1.73) | 7 | 0.46 | (0.18-0.94) | 15 | 0.92 | (0.52-1.52) | 16 | 1.30 | (0.74-2.11) |
| Colon (153) | 2 | 0.61 | (0.07-2.20) | 5 | 0.48 | (0.16-1.12) | 12 | 1.08 | (0.56-1.89) | 11 | 1.31 | (0.65-2.35) |
| Rectum (154) | 1 | 0.56 | (0.01-3.10) | 2 | 0.41 | (0.05-1.46) | 3 | 0.58 | (0.12-1.69) | 5 | 1.28 | (0.42-2.98) |
| Non-smoking-related cancers | 28 | 1.42 | (0.99-2.12) | 47 | 0.86 | (0.63-1.14) | 68 | 1.12 | (0.87-1.42) | 51 | 1.07 | (0.80-1.41) |
| Female breast (174) | 12 | 1.49 | (0.77-2.60) | 19 | 0.81 | (0.49-1.27) | 34 | 1.26 | (0.88-1.77) | 22 | 1.04 | (0.65-1.58) |
| Cervix uteri (180) | 1 | 0.58 | (0.01-3.24) | 5 | 1.80 | (0.58-4.19) | 3 | 1.26 | (0.26-3.67) | 2 | 1.34 | (0.16-4.82) |
| Endometrium (182) | 2 | 0.86 | (0.10-3.11) | 0 | 0.00 | (0.00-0.66) | 2 | 0.34 | (0.04-1.22) | 4 | 0.86 | (0.23-2.19) |
| Ovary (183) | 4 | 2.02 | (0.55-5.18) | 7 | 1.46 | (0.59-3.01) | 7 | 1.38 | (0.56-2.85) | 5 | 1.31 | (0.43-3.06) |
| Thyroid gland (193) | 1 | 3.11 | (0.08-17.3) | 1 | 1.37 | (0.03-7.66) | 3 | 3.99 | (0.82-11.7) | 1 | 1.62 | (0.04-9.01) |
| Other non-smoking-related cancers | 8 | 1.52 | (0.65-2.99) | 15 | 0.86 | (0.48-1.42) | 19 | 0.98 | (0.59-1.53) | 17 | 1.08 | (0.63-1.73) |
| **Men** |  |  |  |  |  |  |  |  |  |  |  |  |
| All but non-melanoma skin | 625 | 1.18 | (1.09, 1.28) | 1002 | 1.25 | (1.17, 1.33) | 786 | 1.19 | (1.11, 1.27) | 469 | 1.17 | (1.07, 1.28) |
| Smoking-related cancers | 357 | 1.46 | (1.32, 1.62) | 508 | 1.61 | (1.47, 1.75) | 362 | 1.53 | (1.38, 1.70) | 227 | 1.81 | (1.58, 2.06) |
| Head and neck | 65 | 1.84 | (1.42, 2.34) | 138 | 2.83 | (2.38, 3.35) | 88 | 2.37 | (1.90, 2.92) | 53 | 2.75 | (2.06, 3.59) |
| Oesophagus (150) | 18 | 1.53 | (0.91, 2.42) | 28 | 1.56 | (1.03, 2.25) | 29 | 2.02 | (1.35, 2.90) | 18 | 2.22 | (1.31, 3.50) |
| Bladder and kidney | 151 | 2.24 | (1.89, 2.62) | 187 | 1.81 | (1.56, 2.08) | 131 | 1.58 | (1.32, 1.88) | 96 | 2.01 | (1.63, 2.45) |
| Leukemias (204-208) | 13 | 0.68 | (0.36, 1.17) | 21 | 0.80 | (0.50, 1.22) | 29 | 1.48 | (0.99, 2.13) | 11 | 1.05 | (0.53, 1.88) |
| Other smoking-related cancers | 110 | 1.00 | (0.82, 1.20) | 134 | 1.12 | (0.94, 1.32) | 85 | 1.03 | (0.83, 1.28) | 49 | 1.23 | (0.9, 1.63) |
| Colorectal (153, 154) | 84 | 1.00 | (0.80-1.24) | 145 | 1.08 | (0.91-1.27) | 106 | 0.97 | (0.80-1.18) | 60 | 0.95 | (0.73-1.23) |
| Colon (153) | 44 | 0.98 | (0.72-1.32) | 87 | 1.12 | (0.90-1.38) | 56 | 0.87 | (0.66-1.13) | 36 | 0.96 | (0.68-1.33) |
| Rectum (154) | 40 | 1.02 | (0.73-1.39) | 58 | 1.03 | (0.78-1.33) | 50 | 1.12 | (0.83-1.48) | 24 | 0.94 | (0.60-1.40) |
| Non-smoking-related cancers | 184 | 0.92 | (0.79-1.06) | 349 | 0.99 | (0.89-1.10) | 318 | 1.01 | (0.90-1.12) | 182 | 0.86 | (0.74-0.99) |
| Prostate (185) | 123 | 1.05 | (0.88-1.26) | 250 | **1.20** | **(1.06-1.36)** | 207 | 1.06 | (0.92-1.21) | 114 | 0.83 | (0.69-1.00) |
| Testis (186) | 2 | 1.46 | (0.18-5.28) | 0 | 0.00 | (0.00-2.51) | 3 | 2.78 | (0.57-8.11) | 0 | 0.00 | (0.00-5.78) |
| Thyroid gland (193) | 3 | 1.23 | (0.25-3.61) | 2 | 0.69 | (0.08-2.49) | 3 | 1.37 | (0.28-3.99) | 2 | 1.63 | (0.20-5.88) |
| Other non-smoking-related cancers | 56 | 0.71 | (0.53-0.92) | 97 | **0.70** | **(0.56-0.85)** | 105 | 0.89 | (0.73-1.08) | 66 | 0.91 | (0.71-1.16) |

Appendix Table 3. Continued

|  | **Small cell carcinoma** | | | | | | | | | | | |
| --- | --- | --- | --- | --- | --- | --- | --- | --- | --- | --- | --- | --- |
| Period at first cancer registration | < 1975 | | | 1975 - 1983 | | | 1984 - 1990 | | | 1991 + | | |
| *Cancer sites (ICD 9th.revision)* | Obs | **SIR** | (95% CI) | Obs | **SIR** | (95% CI) | Obs | **SIR** | (95% CI) | Obs | **SIR** | (95% CI) |
| **Women** |  |  |  |  |  |  |  |  |  |  |  |  |
| All but non-melanoma skin | 14 | 1.78 | (0.97, 2.98) | 46 | 1.41 | (1.03, 1.88) | 51 | 1.10 | (0.82, 1.45) | 38 | 0.88 | (0.62, 1.21) |
| Smoking-related cancers | 6 | 3.25 | (1.19, 7.08) | 18 | 2.69 | (1.59, 4.25) | 22 | 2.47 | (1.55, 3.74) | 12 | 1.52 | (0.78, 2.65) |
| Head and neck | 1 | 6.37 | (0.08, 35.5) | 2 | 2.78 | (0.31, 10.1) | 4 | 3.91 | (1.05, 10.0) | 2 | 2.12 | (0.24, 7.65) |
| Oesophagus (150) | 0 | 0.00 | (0.00, 33.8) | 4 | 7.69 | (2.10, 19.7) | 2 | 3.26 | (0.39, 11.8) | 0 | 0.00 | (0.00, 6.40) |
| Bladder and kidney | 1 | 2.49 | (0.03, 13.9) | 5 | 2.70 | (0.87, 6.30) | 8 | 3.04 | (1.31, 5.98) | 4 | 1.65 | (0.44, 4.22) |
| Leukemias (204-208) | 2 | 9.53 | (1.15, 34.4) | 2 | 2.58 | (0.31, 9.33) | 3 | 2.86 | (0.59, 8.36) | 2 | 2.21 | (0.27, 7.99) |
| Other smoking-related cancers | 2 | 2.07 | (0.23, 7.46) | 5 | 1.76 | (0.57, 4.12) | 5 | 1.39 | (0.45, 3.25) | 4 | 1.31 | (0.35, 3.36) |
| Colorectal (153, 154) | 0 | 0.00 | (0.00-2.98) | 3 | 0.56 | (0.11-1.62) | 5 | 0.67 | (0.22-1.55) | 6 | 0.87 | (0.32-1.90) |
| Colon (153) | 0 | 0.00 | (0.00-4.52) | 2 | 0.55 | (0.07-1.97) | 4 | 0.78 | (0.21-2.00) | 3 | 0.64 | (0.13-1.86) |
| Rectum (154) | 0 | 0.00 | (0.00-8.79) | 1 | 0.58 | (0.01-3.21) | 1 | 0.42 | (0.01-2.33) | 3 | 1.39 | (0.29-4.06) |
| Non-smoking-related cancers | 8 | 1.66 | (0.72-3.28) | 25 | 1.21 | (0.78-1.79) | 24 | 0.80 | (0.52-1.20) | 20 | 0.71 | (0.43-1.09) |
| Female breast (174) | 4 | 2.04 | (0.56-5.23) | 5 | 0.56 | (0.18-1.32) | 10 | 0.74 | (0.35-1.36) | 10 | 0.75 | (0.36-1.38) |
| Cervix uteri (180) | 2 | 6.30 | (0.76-22.8) | 2 | 1.76 | (0.21-6.34) | 2 | 1.47 | (0.18-5.32) | 0 | 0.00 | (0.00-3.62) |
| Endometrium (182) | 0 | 0.00 | (0.00-6.80) | 0 | 0.00 | (0.00-1.84) | 1 | 0.33 | (0.01-1.83) | 2 | 0.73 | (0.09-2.63) |
| Ovary (183) | 0 | 0.00 | (0.00-8.47) | 1 | 0.57 | (0.01-3.19) | 5 | 1.99 | (0.65-4.65) | 3 | 1.28 | (0.26-3.75) |
| Thyroid gland (193) | 0 | 0.00 | (0.00-39.2) | 3 | **10.90** | **(2.26-31.9)** | 0 | 0.00 | (0.00-10.0) | 0 | 0.00 | (0.00-9.19) |
| Other non-smoking-related cancers | 2 | 1.37 | (0.15-4.95) | 14 | 2.13 | (1.17-3.58) | 6 | 0.66 | (0.24-1.44) | 5 | 0.59 | (0.19-1.38) |
| **Men** |  |  |  |  |  |  |  |  |  |  |  |  |
| All but non-melanoma skin | 36 | 1.09 | (0.76, 1.50) | 136 | 1.32 | (1.11, 1.56) | 145 | 1.18 | (1.00, 1.39) | 91 | 0.97 | (0.78, 1.19) |
| Smoking-related cancers | 19 | 1.19 | (0.72, 1.86) | 64 | 1.55 | (1.19, 1.98) | 64 | 1.40 | (1.08, 1.78) | 39 | 1.27 | (0.90, 1.74) |
| Head and neck | 2 | 0.79 | (0.09, 2.85) | 8 | 1.15 | (0.49, 2.26) | 16 | 1.99 | (1.14, 3.24) | 7 | 1.33 | (0.53, 2.73) |
| Oesophagus (150) | 0 | 0.00 | (0.00, 3.81) | 5 | 2.09 | (0.68, 4.88) | 4 | 1.44 | (0.39, 3.70) | 3 | 1.43 | (0.30, 4.19) |
| Bladder and kidney | 9 | 2.67 | (1.22, 5.07) | 26 | 2.01 | (1.31, 2.95) | 23 | 1.49 | (0.94, 2.23) | 12 | 1.06 | (0.55, 1.85) |
| Leukemias (204-208) | 0 | 0.00 | (0.00, 3.30) | 4 | 1.17 | (0.32, 3.00) | 5 | 1.32 | (0.43, 3.08) | 5 | 2.00 | (0.65, 4.67) |
| Other smoking-related cancers | 8 | 1.01 | (0.43, 1.99) | 21 | 1.35 | (0.83, 2.06) | 16 | 1.02 | (0.58, 1.65) | 12 | 1.26 | (0.65, 2.20) |
| Colorectal (153, 154) | 5 | 1.13 | (0.37-2.65) | 18 | 1.03 | (0.61-1.63) | 13 | 0.62 | (0.33-1.06) | 10 | 0.64 | (0.31-1.18) |
| Colon (153) | 3 | 1.24 | (0.26-3.62) | 11 | 1.09 | (0.54-1.94) | 8 | 0.65 | (0.28-1.27) | 7 | 0.76 | (0.30-1.56) |
| Rectum (154) | 2 | 1.01 | (0.12-3.64) | 7 | 0.96 | (0.39-1.98) | 5 | 0.58 | (0.19-1.36) | 3 | 0.48 | (0.10-1.39) |
| Non-smoking-related cancers | 12 | 0.94 | (0.48-1.64) | 54 | 1.22 | (0.92-1.60) | 68 | 1.21 | (0.83-1.31) | 42 | 0.88 | (0.63-1.19) |
| Prostate (185) | 10 | 1.42 | (0.68-2.62) | 37 | **1.53** | **(1.08-2.11)** | 40 | 1.23 | (0.94-1.54) | 24 | 0.80 | (0.51-1.19) |
| Testis (186) | 0 | 0.00 | (0.00-52.1) | 0 | 0.00 | (0.00-15.1) | 1 | 3.66 | (0.09-20.4) | 0 | 0.00 | (0.00-17.7) |
| Thyroid gland (193) | 0 | 0.00 | (0.00-24.0) | 2 | 5.03 | (0.61-18.2) | 1 | 2.21 | (0.06-12.3) | 1 | 3.21 | (0.08-17.9) |
| Other non-smoking-related cancers | 2 | 0.36 | (0.04-1.30) | 15 | 0.78 | (0.43-1.28) | 26 | 1.14 | (0.74-1.67) | 17 | 0.98 | (0.57-1.57) |

Appendix Table 3. Continued

|  | **Adenocarcinoma excluding beonchioalveolar** | | | | | | | | | | | | | | | |
| --- | --- | --- | --- | --- | --- | --- | --- | --- | --- | --- | --- | --- | --- | --- | --- | --- |
| Period at first cancer registration | <1975 | | | | 1975-1983 | | | | 1984-1990 | | | | 1991+ | | | |
| *Cancer sites (ICD 9th.revision)* | Obs | **SIR** | (95% CI) | | Obs | **SIR** | (95% CI) | | Obs | **SIR** | (95% CI) | | Obs | **SIR** | (95% CI) | |
| **Women** |  |  |  |  |  |  |  |  |  |  |  |  |  |  |  |  |
| All but non-melanoma skin | 82 | 1.48 | (1.17, 1.83) | | 162 | 1.67 | (1.42, 1.95) | | 169 | 1.44 | (1.23, 1.67) | | 142 | 1.40 | (1.18, 1.65) | |
| Smoking-related cancers | 24 | 1.95 | (1.25, 2.89) | | 52 | 2.61 | (1.95, 3.42) | | 51 | 2.24 | (1.67, 2.94) | | 37 | 2.02 | (1.42, 2.78) | |
| Head and neck | 2 | 2.05 | (0.23, 7.40) | | 9 | 4.62 | (2.11, 8.77) | | 1 | 0.41 | (0.01, 2.27) | | 2 | 0.97 | (0.11, 3.51) | |
| Oesophagus (150) | 0 | 0.00 | (0.00, 6.66) | | 4 | 3.53 | (0.95, 9.03) | | 3 | 2.27 | (0.46, 6.64) | | 0 | 0.00 | (0.00, 3.50) | |
| Bladder and kidney | 14 | 4.55 | (2.49, 7.64) | | 17 | 3.19 | (1.86, 5.11) | | 21 | 3.18 | (1.97, 4.86) | | 17 | 3.03 | (1.76, 4.85) | |
| Leukemias (204-208) | 1 | 0.71 | (0.01, 3.96) | | 7 | 2.94 | (1.18, 6.06) | | 9 | 3.29 | (1.50, 6.25) | | 5 | 2.21 | (0.71, 5.17) | |
| Other smoking-related cancers | 7 | 1.11 | (0.44, 2.28) | | 15 | 1.64 | (0.92, 2.70) | | 17 | 1.76 | (1.02, 2.82) | | 13 | 1.76 | (0.94, 3.01) | |
| Colorectal (153, 154) | 10 | 1.16 | (0.56, 2.13) | | 20 | 1.29 | (0.79, 1.99) | | 24 | 1.29 | (0.83, 1.92) | | 20 | 1.28 | (0.78, 1.97) | |
| Colon (153) | 7 | 1.24 | (0.50, 2.56) | | 18 | 1.74 | (1.03, 2.75) | | 15 | 1.20 | (0.67, 1.97) | | 16 | 1.51 | (0.86, 2.45) | |
| Rectum (154) | 3 | 1.01 | (0.20, 2.96) | | 2 | 0.38 | (0.04, 1.38) | | 9 | 1.48 | (0.67, 2.81) | | 4 | 0.79 | (0.21, 2.01) | |
| Non-smoking-related cancers | 48 | 1.39 | (1.02, 1.84) | | 90 | 1.46 | (1.18, 1.80) | | 94 | 1.23 | (1.00, 1.51) | | 85 | 1.26 | (1.00, 1.55) | |
| Female breast (174) | 21 | 1.47 | (0.91, 2.25) | | 44 | 1.64 | (1.19, 2.20) | | 38 | 1.09 | (0.77, 1.49) | | 32 | 1.01 | (0.69, 1.42) | |
| Cervix uteri (180) | 2 | 0.74 | (0.08, 2.68) | | 4 | 1.26 | (0.34, 3.24) | | 3 | 0.96 | (0.19, 2.80) | | 3 | 1.36 | (0.27, 3.97) | |
| Endometrium (182) | 3 | 0.79 | (0.16, 2.31) | | 7 | 1.06 | (0.42, 2.18) | | 9 | 1.15 | (0.53, 2.18) | | 0 | 0.00 | (0.00, 0.54) | |
| Ovary (183) | 3 | 0.89 | (0.18, 2.59) | | 4 | 0.74 | (0.20, 1.89) | | 10 | 1.61 | (0.77, 2.96) | | 11 | 2.05 | (1.02, 3.67) | |
| Thyroid gland (193) | 3 | 4.89 | (0.98, 14.3) | | 3 | 3.05 | (0.61, 8.91) | | 6 | 5.35 | (1.95, 11.6) | | 6 | 6.11 | (2.23, 13.3) | |
| Other non-smoking-elated cancers | 16 | 1.63 | (0.93, 2.65) | | 28 | 1.52 | (1.01, 2.20) | | 28 | 1.22 | (0.81, 1.77) | | 33 | 1.61 | (1.11, 2.27) | |
| **Men** |  |  |  |  |  |  |  |  |  |  |  |  |  |  |  |  |
| All but non-melanoma skin | 141 | 1.46 | (1.23, 1.72) | | 281 | 1.40 | (1.24, 1.58) | | 323 | 1.49 | (1.33, 1.67) | | 267 | 1.51 | (1.34, 1.71) | |
| Smoking-related cancers | 59 | 1.41 | (1.07, 1.82) | | 119 | 1.54 | (1.28, 1.84) | | 156 | 2.07 | (1.76, 2.42) | | 94 | 1.76 | (1.42, 2.16) | |
| Head and neck | 11 | 1.73 | (0.86, 3.09) | | 15 | 1.21 | (0.68, 1.99) | | 33 | 2.62 | (1.80, 3.68) | | 19 | 2.20 | (1.32, 3.43) | |
| Oesophagus (150) | 3 | 1.49 | (0.30, 4.34) | | 4 | 0.95 | (0.25, 2.42) | | 13 | 2.90 | (1.54, 4.97) | | 7 | 2.04 | (0.82, 4.20) | |
| Bladder and kidney | 20 | 1.64 | (1.00, 2.54) | | 45 | 1.79 | (1.31, 2.40) | | 58 | 2.22 | (1.69, 2.87) | | 36 | 1.79 | (1.25, 2.48) | |
| Leukemias (204-208) | 4 | 1.17 | (0.32, 3.01) | | 10 | 1.54 | (0.74, 2.84) | | 12 | 1.88 | (0.97, 3.29) | | 7 | 1.52 | (0.61, 3.13) | |
| Other smoking-related cancers | 21 | 1.17 | (0.72, 1.79) | | 45 | 1.55 | (1.13, 2.08) | | 40 | 1.55 | (1.11, 2.11) | | 25 | 1.51 | (0.98, 2.23) | |
| Colorectal (153, 154) | 14 | 0.92 | (0.50, 1.55) | | 38 | 1.14 | (0.80, 1.56) | | 42 | 1.17 | (0.84, 1.58) | | 35 | 1.26 | (0.88, 1.76) | |
| Colon (153) | 6 | 0.71 | (0.26, 1.54) | | 25 | 1.30 | (0.84, 1.92) | | 29 | 1.38 | (0.92, 1.98) | | 21 | 1.28 | (0.79, 1.96) | |
| Rectum (154) | 8 | 1.18 | (0.51, 2.32) | | 13 | 0.93 | (0.49, 1.58) | | 13 | 0.88 | (0.47, 1.50) | | 14 | 1.24 | (0.68, 2.08) | |
| Non-smoking-related cancers | 68 | 1.71 | (1.33, 2.17) | | 124 | 1.38 | (1.15, 1.65) | | 125 | 1.19 | (0.99, 1.42) | | 138 | 1.45 | (1.22, 1.71) | |
| Prostate (185) | 47 | 2.08 | (1.52, 2.76) | | 80 | 1.51 | (1.20, 1.88) | | 82 | 1.29 | (1.02, 1.60) | | 103 | 1.69 | (1.38, 2.05) | |
| Testis (186) | 0 | 0.00 | (0.00, 12.5) | | 1 | 2.22 | (0.03, 12.3) | | 3 | 6.49 | (1.30, 19.0) | | 1 | 2.62 | (0.03, 14.6) | |
| Thyroid gland (193) | 0 | 0.00 | (0.00, 8.35) | | 4 | 5.16 | (1.39, 13.2) | | 3 | 3.75 | (0.75, 11.0) | | 2 | 3.29 | (0.37, 11.9) | |
| Other non-smoking-related cancers | 21 | 1.29 | (0.80, 1.96) | | 39 | 1.10 | (0.78, 1.50) | | 37 | 0.92 | (0.65, 1.27) | | 32 | 0.96 | (0.65, 1.35) | |
